# Dr. John Richard Devine

**Published:** 1914-08

**Authors:** 


Dr. John Richard Devine, Albany, 1910, died at his home in Utica, March 4, of pneumonia, aged 27.

